# Obstetric anal sphincter injury in adolescent mothers

**DOI:** 10.1186/s12884-021-04045-4

**Published:** 2021-08-18

**Authors:** Henry H. Chill, Michal Lipschuetz, Eyal Atias, Tzvika Shimonovitz, David Shveiky, Gilad Karavani

**Affiliations:** 1 Division of Female Pelvic Medicine and Reconstructive Surgery, Department of Obstetrics and Gynecology, Hadassah Medical Organization and Faculty of Medicine, Hebrew University of Jerusalem, Jerusalem, Israel; 2 Division of Obstetrics and Gynecology, Hadassah Medical Organization and Faculty of Medicine, Hebrew University of Jerusalem, Jerusalem, Israel

**Keywords:** Perineal lacerations, Adolescent pregnancy, Vaginal tears, Obstetric anal sphincter injury

## Abstract

**Background:**

Obstetric anal sphincter injury (OASI) is a debilitating complication of vaginal delivery which has yet to receive ample attention in adolescents. The aim of this study was to describe risk for OASI in adolescent mothers compared to adults. We further attempted to compare risk factors for OASI between these two age groups.

**Methods:**

We performed a retrospective cohort study between 2003 and 2019. Primiparous women who delivered vaginally, 21 years and younger were compared to women ages 26–35. Excluded were preterm, multifetal, non-vertex, cesarean deliveries as well as intrauterine fetal death. Rate of OASI as well as obstetric and labor characteristics of women with OASI, were compared between groups. Finally, risk factors were assessed for each group separately. Univariate and multivariate logistic regression model were performed.

**Results:**

Final analysis was performed on 5113 nulliparous adolescents and 13,845 nulliparous in the 26–35 age group. Allocation to study groups was according to OASI – Sixty-seven adolescents (1.3%) had a 3rd or 4th degree perineal tear and were defined as the OASI group, while 5046 patients (98.7%) did not have such a tear. In the adult group, 199 out of 13,845 patients (1.4%) were diagnosed with OASI. Occurrence of OASI did not differ between groups (*p* = 0.510). Comparison of women with OASI in the adolescent group vs. adult group found differences with regard to operative vaginal delivery, (20.9% vs. 36.2%, respectively; *p* = 0.023) and meconium stained amniotic fluid (9.1% vs. 21.3%, respectively; *p* = 0.027).

Following multivariate analysis the only parameter independently associated with OASI in the adolescent age group was head circumference ≥ 90th percentile with an adjusted odds ratio of 3.08 (CI 1.48–6.38, *p* = 0.003). In the adult group the similar analysis revealed operative vaginal delivery (OR = 2.44, CI 1.72–3.47, *p* < 0.001) and a birthweight≥90th percentile (OR = 2.23, CI 1.19–4.18, *p* = 0.012) to be independent risk factors for OASI.

**Conclusion:**

Adolescents have similar risk for OASI compared to adults but differ in risk factors leading to OASI. Head circumference ≥ 90th percentile was found to be associated with OASI in this age group.

## Background

Adolescent pregnancy is a phenomenon estimated to occur in 13% of women in the United States and 25% worldwide [1, 2]. Though steadily declining over the past decade, the impact of adolescent pregnancies on health outcomes is too significant to go overlooked [3]. Currently, complications during pregnancy and childbirth are the second leading cause of mortality in the 15 to 19-year-old age group worldwide [4].

Several studies have presented outcomes of pregnancy in adolescent mothers. While most report on increased risk of preterm delivery, data regarding other complications of pregnancy and childbirth have been equivocal [5–7].

One complication which has yet to receive ample attention in adolescents is obstetric anal sphincter injury (OASI). While being a debilitating complication of vaginal delivery, data regarding risk factors for OASI in this population are sparse. Some studies have reported on similar risk of perineal lacerations in adolescents while others have suggested young age to be a protective factor with respect to such injury [8, 9].

Risk factor such as operative vaginal delivery, increased birth weight, increased head circumference, prolonged second stage of labor and advanced maternal age have previously been associated with OASI but have not been thoroughly assessed in adolescents [10–13]. In addition we have previously described an association between large fetal and neonatal anthropomorphic measures and obstetric outcomes. These findings focused primarily on the large head circumference (HC) which was found associated with higher rates of interventional deliveries (cesarean, vacuum, failed vacuum, deliveries) [14–16] and higher rates of persistent occiput posterior position [17].

More recently, data has accumulated with respect to sonographic diagnosis of intrapartum sphincter tears in primiparous women showing a correlation between sonographic demonstration of tear characteristic signs and Levator Ani muscle tears [18, 19] and clinical complaints of incontinence [20].

The aim of this study was to describe risk for OASI in adolescent mothers compared to adults. We further attempted to compare risk factors for OASI between these two age groups.

## Methods

We performed a retrospective cohort study at a tertiary, university, teaching medical center, between 2003 and 2019. Our cohort was comprised of primiparous women who delivered vaginally during the study period. Excluded were preterm, non-vertex, multifetal, cesarean deliveries as well as intrauterine fetal death. Institutional review board approval was received (IRB 0348–20-HMO).

The study group included women following vaginal delivery up to the age of 21. This cutoff for adolescence has been endorsed by the US department of health and the American academy of Pediatrics [21, 22]. Obstetric anal sphincter injury rate in this group was compared to occurrence of OASI in women ages 26–35. This age group was chosen as the reference group so as to distance it from younger age groups as well as older women over the age of 35 for which the process of labor may be somewhat different. Women ages 22–25 years, categorized by some as young adults, were excluded so as to eliminate any effect this special subgroup may have on the results [23]. For the purpose of assessing specific risk factors for OASI according to age, women with OASI were compared to women without such injury in each age group separately.

Data was collected from the delivery-room electronic medical records. Parameters evaluated included age, parity, comorbidities, mode of previous delivery, obstetric history, gestational week at delivery, method of labor initiation, prolonged second stage, mode of delivery, episiotomy, neonatal head circumference (HC) and birth weight (BW).

Laceration severity was categorized according to the American College of Obstetricians and Gynecologists practice bulletin [24]. As described by us previously, OASI was diagnosed by the most senior physician on the labor and delivery floor. Once diagnosis was established the patient was transferred to the operating room where the anal sphincter was repaired.

### Statistical analysis

Comparison was performed between parturients with OASI aged ≤21 and ages 26–35. In addition, in every age group, we compared those with and without OASI to identify risk factors associated with each group. Categorical variables were assessed using the Chi-square and Fischer’s exact tests for small numbers, as appropriate. The t-test and Mann-Whitney U tests were implemented for continuous variables. Following univariate analysis, for each age group a multivariate analysis using logistic regression was performed. We report adjusted odds ratios (aOR), 95% confidence interval (CI) for parameters included in the final multivariate analysis. Analyses were two-tailed and *p*-values of < 0.05 were considered significant. Statistical analysis in this study was performed using Office Excel 2010 (Microsoft, Seattle, WA) and IBM SPSS 27 for Windows (IBM corp. Armonk, NY).

## Results

**Fig. 1:**
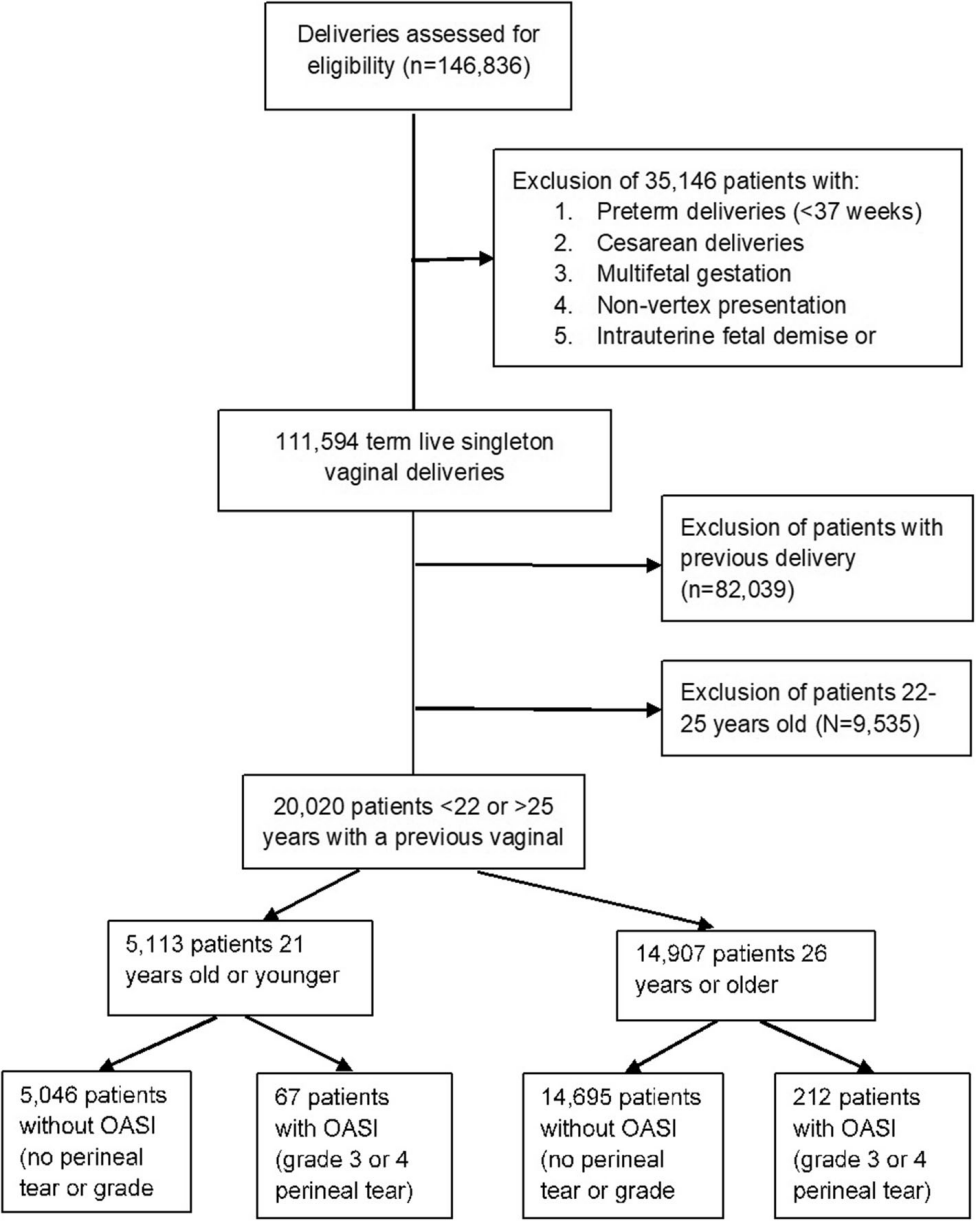
Study population flow chart

During the study period, 146,386 patients delivered in our institution and were evaluated for eligibility. Following implementation of the exclusion criteria, 111,594 patients with a live, singleton vaginal delivery remained. We further excluded multipara parturients and those who were 22–25 or older than 35 years (92,636), focusing on women 21 years and under (adolescent group) and women 26–35 years of age (adult group) (Fig. 1). Finally, 5113 nulliparous adolescents and 13,845 women in the control group were included in the analysis. Allocation to study groups was according to OASI – Sixty-seven adolescents (1.3%) had a 3rd or 4th degree perineal tear and were defined as the OASI group, while 5046 patients (98.7%) did not have such a tear i.e. “no OASI” group. In the adult group, 199 out of 13,845 patients (1.4%) were diagnosed with OASI (Fig. 1). The occurrence of OASI did not differ between age groups (*p* = 0.510).

Table 1 presents the comparison of obstetric and labor related characteristics of patients with obstetric anal sphincter injury (OASI) comparing the adolescents (*n* = 67) to the adult age group (*n* = 212). The only parameters that differed significantly between adolescent and adult groups were rates of operative vaginal delivery (20.9% vs. 36.2%, respectively; *p* = 0.023) and meconium-stained amniotic fluid (9.1% vs. 21.3%, respectively; *p* = 0.027).

**Table 1:** Obstetric and labor related characteristics of patients with obstetric anal sphincter injury (OASI) according to age groups

Parameter	≤21 years	26–35 years	***P*** value^**a**^
**No. of patients**	67	199	
**Gestational diabetes**	0	6 (3.0%)	0.342
**PIH/Preeclampsia**	2 (3.0%)	0	0.063
**Gestational week**	39.8 ± 1.1 (40)	39.7 ± 1.1 (40)	0.386
**Induction of labor**	15 (22.4%)	59(29.6%)	0.274
**Epidural analgesia**	46 (69.7%)	151 (76.3%)	0.328
**Artificial rupture of membranes**	29/58 (50.0%)	85/172 (49.4%)	1.000
**Meconium stained amniotic fluid**	6 (9.1%)	42 (21.3%)	0.027
**Prolonged 2nd stage**	14 (20.9%)	50/194 (25.1%)	0.511
**Operative vaginal delivery**	14 (20.9%)	72 (36.2%)	0.023
**Birth weight (grams)**	3385 ± 421 (3406)	3381 ± 413(3386)	0.950
**Birth weight > 90th percentile (> 3900 g)**	5 (7.5%)	19 (9.5%)	0.806
**Head circumference (cm)**^b^	34.5 ± 1.4 (34.3)	34.6 ± 1.2 (34.5)	0.846
**Head circumference > 90th percentile (> 36 cm)**^b^	12/51 (23.5%)	23/139 (16.5%)	0.294
**Episiotomy**	18 (26.9%)	67 (33.7%)	0.364

Data presented as mean ± SD (median) or n(%) or n/N (%)

^a^ Calculated using Chi square test/ Fisher exact test or Mann-Whitney test

^b^Electronic records available since 2010 for 51 in the adolescent group and for 149 in the 26–35 age group

We further focused on each age group - adolescents and adults comparing patients with and without OASI in each age group. The descriptive analysis of the adolescent and the adult group population according to OASI is presented in Table 2. In the adolescent age group, no differences were noted upon comparison of rates of induction of labor (22.4% vs. 15.3%, respectively; *p* = 0.079), epidural analgesia (69.7% vs. 64.5%, respectively; *p* = 0.438), artificial rupture of membranes (50.0% vs. 54.2%, respectively; *p* = 0.596), prolonged second stage of labor (20.9% vs. 14.4%, respectively; *p* = 0.160) and operative vaginal delivery (20.9% vs. 15.2%, respectively; *p* = 0.23). Evaluation of neonatal parameters in the OASI vs. no OASI groups demonstrated higher BW (3385 ± 421 vs. 3321 ± 397 g, respectively; *p* = 0.001), larger HC (34.5 ± 1.4 vs. 34.2 ± 1.2 cm, respectively; *p* = 0.047) and increased rate of HC above 90th percentile (24.1% vs. 9.1%, respectively; *p* = 0.002) in the OASI group.

**Table 2:** Obstetric and labor related characteristics of patients with and without obstetric anal sphincter injury (OASI) according to age groups

	≤21 years			26–35 years		
Parameter	OASI	No OASI	*P* value^**a**^	OASI	No OASI	*P* value^**a**^
**No. of patients**	67 (1.3%)	5046 (98.7%)		199 (1.7%)	13,646(98.3%)	
**Age**	20.2 ± 1.0 (21.0)	20.3 ± 1.0 (20.6)	0.780	28.8 ± 2.3 (28.0)	29.2 ± 1.2 (29.0)	0.008
**Gestational diabetes**	0	32 (0.6%)	1.000	6 (3.0%)	263 (1.9%)	0.289
**PIH/Preeclampsia**	2 (3.0%)	5/4342 (1.2%)	0.198	0	107/11662 (1.2%)	0.430
**Gestational week**	39.8 ± 1.1 (40)	39.6 ± 1.2 (40)	0.079	39.7 ± 1.2 (40)	39.7 ± 1.1 (40)	0.007
**Induction of labor**	15 (22.4%)	769 (15.3%)	0.082	59 (29.6%)	3089 (22.7%)	0.025
**Epidural analgesia**	46 (69.7%)	3258 (64.5%)	0.438	151 (76.3%)	10,358 (75.9%%)	0.994
**Artificial rupture of membranes**	29/58 (50.0%)	2190/4042(54.2%)	0.596	85/172 (49.4%)	5319/11,074(48.0%)	0.759
**Meconium stained amniotic fluid**	6 (9.1%)	758/4860 (15.6%)	0.172	42 (21.3%)	2496 (18.9%)	0.412
**Prolonged 2nd stage**	14 (20.9%)	714/4947 (14.4%)	0.160	50 (25.8%)	2847 (21.3%)	0.132
**Operative vaginal delivery**	14 (20.9%)	769 (15.2%)	0.229	72 (36.2%)	3044 (22.3%)	< 0.001
**Birth weight (grams)**	3385 ± 421 (3406)	3321 ± 397 (3214)	0.001	3382 ± 413 (3386)	3205 ± 395(3190)	< 0.001
**Birth weight > 90th percentile (> 3900 g)**	5 (7.5%)	240 (4.7%)	0.252	19 (9.5%)	603 (4.4%)	0.003
**Head circumference (cm)**^b^	34.5 ± 1.4 (34.3)	34.2 ± 1.2 (34.0)	0.047	34.6 ± 1.2 (34.5)	34.3 ± 1.2 (34.3)	0.007
**Head circumference > 90th percentile (> 36 cm)**^b^	12/51 (24.1%)	210/2317 (9.1%)	0.002	23/139 (16.5%)	733/7138 (10.3%)	0.024
**Episiotomy**	18 (26.9%)	1245/4340(28.7%)	0.892	67 (33.7%)	2908 (24.9%)	0.006

Data presented as mean ± SD (median) or n(%) or n/N (%). ^a^ Calculated using Chi square test/ Fisher exact test or Mann-Whitney test. ^b^Electronic records available since 2010 for 51 in OASI group and for 2317 in the remaining cohort

In the adult group, higher rates of induction of labor and operative vaginal delivery were demonstrated in the OASI vs. no OASI group (29.6% vs. 22.7 and 36.2% vs. 22.3%, *p* = 0.025 and *p* < 0.001; respectively). Neonatal characteristics – higher mean neonatal BW and HC as well as the rates of neonates with larger than 90th percentile HC and BW were also shown in the OASI group (Table 2). Finally, women with OASI had more episiotomies than those without OASI (33.7% vs. 24.9%, respectively; *p* = 0.006).

Multivariate regression models were applied to identify factors associated with OASI for the adolescent and adult groups separately (Table 3). The model included age, prolonged second stage of labor, operative vaginal delivery, BW above 90th percentile and HC above 90th percentile. In the adolescents’ model, the analysis showed that the only parameter independently associated with OASI in the adolescent age group was HC ≥ 90th percentile with an adjusted odds ratio of 3.08 (CI 1.48–6.38, *p* = 0.003).

**Table 3:** Multivariate analysis of parameters associated with obstetric anal sphincter injury

Parameter	OR (95% CI)	*P* value	OR (95% CI)	*P* value
	≤21 years		26–35 years	
**Age**	0.90 (0.69–1.19)	0.461	0.92 (0.85–0.99)	0.018
**Prolonged second stage of labor**	0.79 (0.40–1.59)	0.514	0.79 (0.40–1.59)	0.514
**Operative vaginal delivery**	1.41 (0.72–2.78)	0.316	2.44 (1.72–3.47)	< 0.001
**Birth weight > 90th percentile**	0.90 (0.29–2.80)	0.853	2.23 (1.19–4.18)	0.012
**Head circumference > 90th percentile**	3.08 (1.48–6.38)	0.003	1.27 (0.76–2.12)	0.354

In the adults group model, the analysis revealed operative vaginal delivery (aOR = 2.44, CI 1.72–3.47, *p* < 0.001) and BW ≥ 90th percentile (aOR = 2.23, CI 1.19–4.18, *p* = 0.012) to be independent risk factors for OASI.

**Fig. 2:**
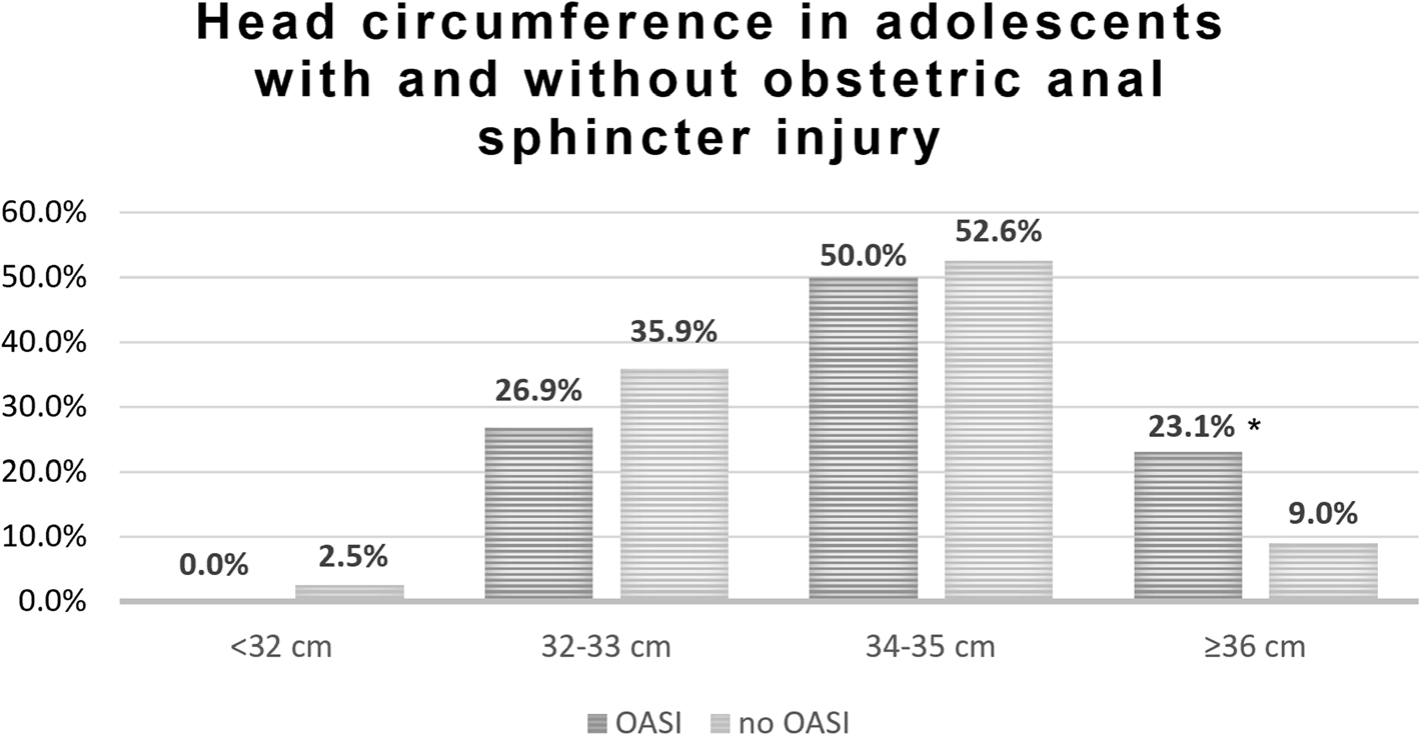
Neonatal head circumference categories in laboring adolescents according to obstetric anal sphincter injury occurrence. *Note:* OASI, obstetric anal sphincter injury, *p* = 0.001*

Adolescent HC (cm) categories in the OASI vs. no OASI groups are presented in Fig. 2. Head circumference categories of under 32 cm, 32–33 cm and 34–35 cm were similar in both groups, while in the category of HC ≥36 cm a significant difference was noted, with a rate of 23.1% in the OASI group vs. 9.0% in the group of adolescents with no OASI (*p* = 0.001)

## Discussion

This study focuses on OASI in adolescents attempting to describe the unique characteristics of this important group. We found similar rates of OASI between adolescent parturiets and the adult group. Comparison between the groups revealed adolescents underwent substantially lower rate of operative vaginal delivery. We further found increased BW, increased HC and HC ≥ 90th percentile to be risk factors for OASI within this subgroup. Following logistic regression, only HC ≥ 90th percentile was found to be an independent risk factor for OASI. In the adult group risk factors for OASI included operative vaginal delivery and BW ≥ 90th percentile.

Previous studies have described risk of OASI in adolescents. Landy et al. analyzed 87,267 deliveries finding OASI rate in women over the age of 25 to be increased compared to women under the age of 25 (OR = 1.6) [25]. In another large cohort, Torvie et al. presented data regarding 26,091 nulliparous adolescents and young adults. They found teens aged 15–19 years had decreased risk of OASI compared to young adults ages 20–24 [26].

A previous analysis performed by us was based on data available from the Consortium on Safe Labor (CSL) database, which included detailed demographic, obstetric and neonatal information from medical records of 228,562 deliveries from 19 hospitals across the U.S. This study evaluated data from 4 MedStar Health hospitals which were considered 1 of 19 medical centers in the CSL and included 203 young adolescents (15 years or younger) and 5753 adolescents (16–21 years) who were compared to the reference group of women ages 22–34. Teenage mothers were 50% less likely to have OASI compared to the control group (adjusted OR = 0.480, CI 0.320–0.720) [27]. In our current study we found a similar rate of OASI in adolescent and adult groups. The differences between our results and the results of the studies mentioned may be explained by the low rate of OASI in our cohort. In a population with lower disposition for OASI, differences between age groups may be less prominent. In addition, the population and the comparison groups differed between studies. For instance, study population of the Consortium on Safe Labor database consisted of a higher proportion of young adolescents (age 15–19), which may have an impact on OASI rate.

Our findings are in accordance with previous studies showcasing most of the known risk factors for OASI discussed in current literature [10–13]. With that, our findings point towards certain differences between the risk factors of adolescents and adults. We found neonatal as opposed to obstetric characteristics to affect risk for OASI in adolescents. Following multivariate analysis, HC ≥ 90th percentile was the sole independent risk factor for OASI in this subgroup. Other previously proven risk factors for OASI such as operative vaginal delivery did not increase risk for OASI in our study, though it may be attributed to the relatively small study group of adolescents with OASI. In contrast, independent risk factors for the adult group included operative vaginal delivery and BW ≥ 90th percentile. One hypothesis could be that the adolescent perineum may have different elastic properties compared to older women. These properties may affect the tissue’s ability to adapt and stretch when confronted with the forces of labor. In this scenario increased HC stretching the perineum may have a detrimental effect on anal sphincter integrity. Another possible explanation for the significant impact of large HC on OASI in adolescents may be that a certain proportion of adolescent mothers have not yet completed their growth. Smaller pelvic diameters may explain the higher susceptibility of adolescent mothers to large HC. This hypothesis remains unsupported as we do not routinely collect pelvimetry data.

Limitations of this study include its retrospective design, the relatively small study group and partially missing data for certain parameters within the cohort. With that being we present one of the largest cohorts to focus on OASI in adolescents.

Data regarding functional outcome following OASI were unavailable. The statistical analysis done for a relatively large number of parameters carries the risk of false positive results due to multiple comparisons.

Few women included were in their teens limiting our ability to assess risk factors in young adolescents.

## Conclusion

In summary we found adolescents have similar risk for OASI compared to older women but differ in risk factors leading to OASI. While HC ≥ 90th percentile was found to be an independent risk factor for such injury other known risk factors for OASI were not found to be significant. Gaining a better understanding of the risk factors and mechanisms of severe perineal lacerations in this subgroup may lead to better clinical care and improved patient outcomes in the future.

## Data Availability

The datasets used and/or analyzed during the current study are available from the corresponding author on reasonable request.

## Abbreviations

OASI: Obstetric anal sphincter injury
HC: head circumference
BW: birth weight
